# A Rare Case of Huge Gastric Adenoma Composed of Pyloric Gland Adenoma and Foveolar-Type Adenoma with Serrated Dysplasia Cured by Endoscopic Submucosal Dissection

**DOI:** 10.5152/tjg.2025.24799

**Published:** 2025-04-21

**Authors:** Shuai Han, Shouxin Yin, Zongguo Sun, Shuangshuang Ren, Zhi Wei

**Affiliations:** 1 Department of Gastroenterology, People’s Hospital Affiliated to Shandong First Medical University, Shandong, China; 2 Department of Pathology, People’s Hospital Affiliated to Shandong First Medical University, Shandong, China; 3 Department of Gastroenterology, Shandong Second Provincial General Hospital, Shandong, China

Dear Editor,

As rare neoplasms, gastric pyloric gland adenomas (PGAs) and foveolar-type adenomas (FTAs) are epithelial polyps composed of neoplastic pyloric-type glands and neoplastic foveolar epithelium, respectively. Gastric serrated dysplasia as a subtype of gastric dysplasia has also been limitedly reported. Herein, the authors present a rare case of huge gastric adenoma composed of PGA and FTA with serrated dysplasia, which was successfully cured by endoscopic resection.

A 52-year-old woman with a history of *Helicobacter pylori* infection but no family history of gastric cancer or polypoid syndromes was admitted due to abdominal discomfort. She was informed and provided written consent for the use of her medical history, current findings, and photographs. An esophagogastroduodenoscopy suggested C-3 type gastric atrophy. In the non-atrophic mucosa, a substantial nodular mass inferior to the cardia was observed (Figure 1A). At the base of the mass, a whitish flat lesion extended into the fundus. The flat elevation resembled a laterally spreading tumor and covered half of the fundus (Figure 1B). Magnifying endoscopy revealed various microsurface and microvascular patterns, suggesting the presence of various tissue structures. Compared with the flat elevation, the nodular mass primarily exhibited pinecone-like microsurface patterns. En-bloc endoscopic submucosal dissection was performed. The specimen fragmented during retrieval. The reassembled specimen measured 16.5 × 14.0 cm. Hematoxylin-eosin staining (Figures 1C and 2A) revealed that the superficial layer of the lesion exhibited neoplastic foveolar-type glands, while the deeper layer showed neoplastic pyloric-type glands with glandular distortion and dilation. The deepest portion contained residual non-neoplastic fundic glands. Immunohistochemical analysis demonstrated that MUC5AC (Figure 2B) was positive in the foveolar-type glands, and MUC6 (Figures 1D and 2C) was positive in the pyloric-type glands. Pepsinogen I, H+/K+ ATPase, CD10, and MUC2 were negative in the neoplastic glands. The final pathological diagnosis confirmed a composite tumor consisting of superficial FTA and deep PGA, exhibiting high-grade dysplasia, along with focal intramucosal well-to-moderately differentiated tubular adenocarcinomas. The amount of residual oxyntic glands at the nodular mass was significantly less than that at the flat elevation. Serrated dysplasia similar to the traditional serrated adenoma of the colorectum was widely present, especially at the nodular mass (Figure 2D). Immunohistochemical analysis revealed no loss of MMR proteins. Remnant or recurrent tumors were not detected at a follow-up time of 1 year.

Sporadic gastric PGAs are usually found within the atrophic or metaplastic corpus mucosa, due to autoimmune or *H*.* pylori* gastritis. In contrast, FTAs and syndromic PGAs occur in the oxyntic gastric mucosa without any inflammation or atrophy/metaplasia. Sporadic FTAs are exceedingly rare, whereas syndromic FTAs are more frequent. This case demonstrates the coexistence of sporadic PGA and FTA within a single lesion in the oxyntic gastric mucosa without atrophy/metaplasia. Reports of such gastric lesions are scarce. In reported cases,the lesions are typically located in the upper two-thirds of the stomach, mainly within non-atrophic regions, presenting as whitish flat elevations.This case aligns well with these findings but is larger (possibly the largest reported of this type) and more complex, presenting as a mixture of laterally spreading tumor and large nodular mass. Histologically, the lesion exhibits a replacement growth pattern, with the base retaining varying amounts of normal tissue structure; the superficial layer is an FTA, and the deeper layer is predominantly a PGA. This histopathological complexity directly correlates with the various endoscopic presentation. PGAs located in fundic gland mucosa rather than pyloric gland mucosa may be related to the proliferation and differentiation of mucous neck cells. Therefore, gastric PGAs combined with FTAs can be considered tumors differentiating into foveolar epithelial cells and mucous neck cells. Genetic studies on such lesions remain limited. Naka T et al^1^ reported two cases sharing *APC* and *KRAS* mutations between the FTA and PGA components, while *GNAS* mutations were exclusive to the PGA component. Although genetic testing was not performed in this case, further studies on genetic alterations in similar lesions are warranted.

It is notable that the lesion exhibited pronounced serrated architecture, with the nodular elevated parts resembling colorectaltraditional serrated adenomas. While colorectal serrated lesions have been extensively studied, gastric serrated lesions or dysplasia remain poorly understood. A review of various reports suggests that serrated adenomas are mostly located in the upper two-thirds of the stomach, appearing as flat or large nodular elevations, with nearly a 70% carcinoma incidence.^2^ In this case, although the lesion is large and structurally complex, the carcinoma is still confined to the mucosa. Therefore, endoscopic resection of lesions is advocated for when the likelihood of cure is high to preserve stomach integrity. Regarding immunophenotypic and molecular features, Hasuo et al^3^ reported a case where the serrated lesion demonstrated wild-type *p53*, stable microsatellites, and no mutations of *BRAF* (exon 11 and 15) and *K-ras* (exon 2 and 3). Kwon et al^4^ reported 9 cases of gastric serrated lesions, with 3 cases showing *KRAS* mutations and no *BRAF* mutations and 2 cases with MLH1 expression loss. Zhu et al^5^ described a case and identified a mutation in exon 2 of the *KRAS* gene, with no mutations in *NRAS*, *PIK3CA*, or *BRAF* genes. Further research is needed on the genetic alterations and pathogenesis of gastric serrated dysplasia.

In summary, a rare and clinically significant case of huge gastric adenoma composed of PGA and FTA with serrated dysplasia is presented, which was successfully cured by endoscopic resection. This case highlights the importance of early detection and the efficacy of endoscopic submucosal dissection as a curative approach for such complex lesions. More researches on the clinicopathological and genetic characteristics of similar cases are warranted to enhance the understanding and management of these rare gastric neoplasms.

## Figures and Tables

**Figure 1.**
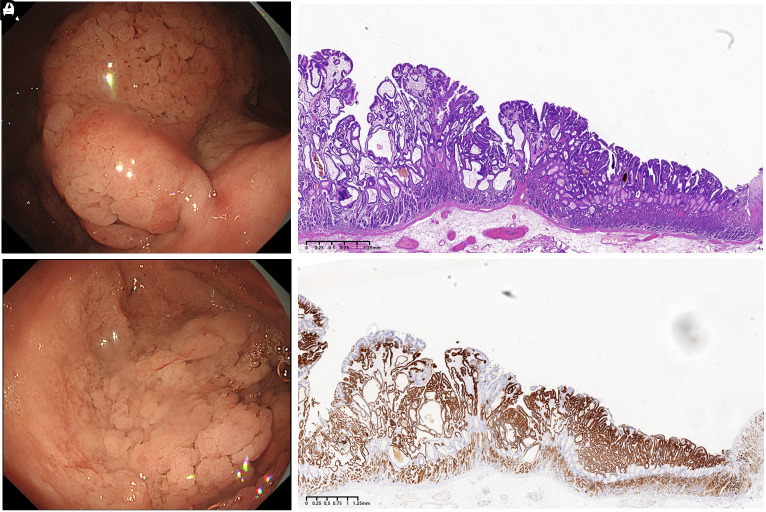
A. Forward observation of the nodular mass inferior to the cardia. B. A flat elevation reminiscent of a laterally spreading tumor occupied half of the fundus. C. Hematoxylin-eosin staining of the flat elevation area. D. MUC6 immunohistochemical staining of the flat elevation area.

**Figure 2.**
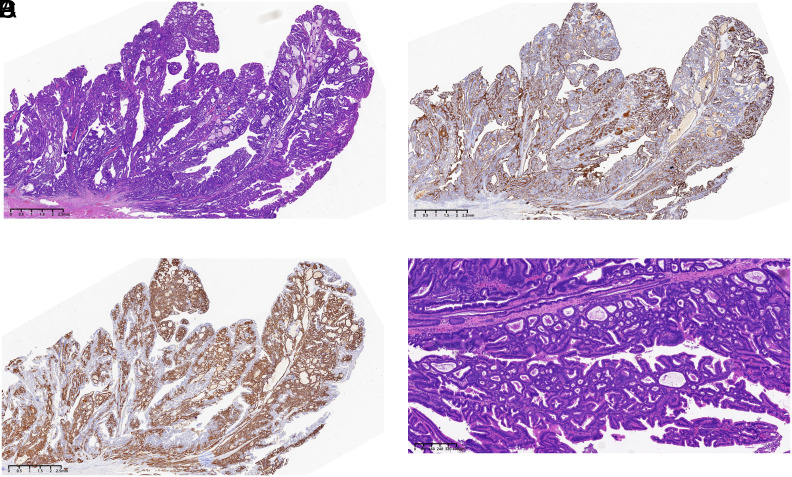
A. Hematoxylin-eosin staining of the nodular mass area. B. MUC5AC immunohistochemical staining of the nodular mass area. C. MUC6 immunohistochemical staining of the nodular mass area. D. Serrated dysplasia similar to the traditional serrated adenoma of colorectum was widely present at the nodular mass.
